# Identification of occult hepatitis B infection despite high anti-HBs in a hemodialysis patient during pretransplant evaluation

**DOI:** 10.1016/j.idcr.2026.e02768

**Published:** 2026-09-12

**Authors:** Bui Thai Huy Nguyen, Hoang Phuong Tran, Nguyen Tra Uyen Ho, Thai Nguyen Ma, Tin Nghia Tran, Quang Khai Tran, Duy Linh Nguyen

**Affiliations:** a Department of Infectious Diseases, Can Tho University of Medicine and Pharmacy, 179 Nguyen Van Cu street, Tan An ward, Can Tho, 900000, Viet Nam; b Department of Biochemistry, Can Tho University of Medicine and Pharmacy, 179 Nguyen Van Cu street, Tan An ward, Can Tho, 900000, Viet Nam; c Department of Pediatrics, Can Tho University of Medicine and Pharmacy, 179 Nguyen Van Cu street, Tan An ward, Can Tho, 900000, Viet Nam; d Department of Surgery, Can Tho University of Medicine and Pharmacy, 179 Nguyen Van Cu street, Tan An ward, Can Tho, 900000, Viet Nam

**Keywords:** Occult hepatitis B infection, Diagnostic pitfall, Hemodialysis, Kidney transplantation, HBV reactivation

## Abstract

Occult hepatitis B virus infection is characterized by detectable hepatitis B virus DNA in individuals who test negative for hepatitis B surface antigen (HBsAg). In patients awaiting kidney transplantation, unrecognized occult hepatitis B virus infection may increase the risk of viral reactivation following immunosuppressive therapy. However, hepatitis B screening in many clinical settings still relies primarily on HBsAg and anti- HBs testing, which may fail to identify occult infection. We report the case of a 39-year- old woman with end-stage renal disease receiving maintenance hemodialysis who was evaluated for kidney transplantation. Routine serological screening showed negative HBsAg and a high anti-HBs titer (292.3 IU/L), suggesting resolved hepatitis B virus infection. However, further evaluation revealed positive total anti-HBc and detectable serum hepatitis B virus DNA with a viral load of 9.54 × 10^3^ lU/mL. Hepatitis B e antigen (HBeAg) was negative and liver enzyme levels were within normal ranges. These findings were consistent with occult hepatitis B virus infection. This case highlights a potential diagnostic pitfall when hepatitis B screening relies solely on HBsAg and anti-HBs testing. A more comprehensive evaluation, including anti-HBc and hepatitis B virus DNA testing, should be considered in high-risk populations to avoid missed occult infection and reduce the risk of hepatitis B virus reactivation after transplantation.

## Highlights

• Occult hepatitis B infection may occur despite high anti-HBs titers.
• Screening based only on HBsAg and Anti-HBs may miss occult HBV infection.
• Hemodialysis patients are at increased risk of occult HBV infection.
• Anti-HBc and HBV DNA testing should be considered during pre-transplant screening.

## Introduction

Hepatitis B virus (HBV) infection remains a major global health problem, with an estimated 296 million people living with chronic HBV infection worldwide [1]. The Vietnamese Ministry of Health estimates that the prevalence of chronic HBV infection in Vietnam ranges from 8% to 25% [2,3]. Chronic HBV infection can lead to serious complications including liver cirrhosis, liver failure, and hepatocellular carcinoma, contributing substantially to global morbidity and mortality [4,5].

In addition to classical chronic hepatitis B characterized by positive hepatitis B surface antigen (HBsAg), another clinical entity that has gained increasing attention is occult hepatitis B virus infection (OBI) [6]. OBI is defined as the presence of HBV DNA in the blood or liver tissue of individuals who test negative for HBsAg, usually accompanied by positive total anti-HBc and with or without anti-HBs antibodies [7]. In kidney transplant recipients, OBI may reactivate following transplantation due to immunosuppressive therapy, potentially resulting in increased HBV DNA levels, reappearance of HBsAg, liver injury, severe hepatitis, or liver failure [8,9]. There are not many statistical studies on the prevalence of OBI in Vietnam. A 2021 study by Tran Thanh Tung et al. found the prevalence of OBI among blood donors to be 2/623 people (0.3%) [8]. Another study by Huynh Hoang Khanh Thu et al. in 2025 found the prevalence of OBI (HBV DNA positive and HBsAg negative) in healthy adults to be 37/397 (9.32%) [10].

Although viral loads in OBI are generally low, this condition has important clinical implications. OBI may lead to viral transmission through blood transfusion or organ transplantation and may reactivate in immunocompromised patients, including those receiving chemotherapy, immunosuppressive therapy, or organ transplantation [11].

Patients with chronic kidney disease, particularly those receiving long-term hemodialysis and awaiting kidney transplantation, represent a high-risk group for HBV infection and reactivation.

A study in Vietnam indicated that the rate of HBsAg positivity in end-stage renal disease patients undergoing hemodialysis is 7% [2]. Repeated invasive procedures, frequent blood transfusions, and impaired immune function associated with renal failure contribute to this increased risk [12,13].

According to the 2019 guidelines for the diagnosis and treatment of hepatitis B virus infection issued by the Vietnamese Ministry of Health, HBV screening at medical facilities only uses the HBsAg test. In cases where patients are prescribed immunosuppressive drugs, chemotherapy, or organ transplantation, HBV screening tests include: HBsAg, anti-HBs, and anti-HBc. [2,14]. However, this strategy may fail to detect OBI, especially in individuals with previous exposure to HBV.

We report a case of a patient with end-stage renal disease with initial negative pre-transplant screening for HBV infection with HBsAg and anti-HBs tests but then was found to have OBI.

## Case presentation

A 39-year-old woman with a history of end-stage renal disease due to congenital right renal atrophy and left ureteral stenosis on maintenance hemodialysis three times per week, was admitted for pre-transplant evaluation as part of the kidney transplantation program. She never received blood transfusions or blood products and had not been vaccinated against hepatitis B. She was admitted for pre-transplant evaluation as part of the kidney transplantation program, including screening for potential infections prior to initiation of immunosuppressive therapy. On physical examination, the patient was alert and hemodynamically stable. No fever, jaundice, hepatosplenomegaly, peripheral edema, or skin hemorrhages were observed. The remainder of the physical examination was unremarkable. Liver function tests including AST, ALT, bilirubin and others were within normal ranges*.*

Routine hepatitis B and hepatitis C screening was initially performed using hepatitis B surface antigen (HBsAg (ELISA)) and anti-HBs (ELISA), anti-HCV (ELISA) testing. Since the initial examining physicians at the outpatient department were not organ transplant specialists, the patient underwent only basic hepatitis B screening using HBsAg and Anti-HBs tests. The results showed negative HBsAg and a high anti- HBs titer of 292.3 IU/L, suggesting resolved hepatitis B virus infection, anti-HCV testing was negative. Based on these findings, the patient was initially considered to have a low risk of HBV reactivation, and antiviral prophylaxis was not considered necessary. However, given the patient's high-risk status as a candidate for kidney transplantation, additional testing including total anti-HBc and hepatitis B e antigen (HBeAg) was performed. The results showed positive total anti-HBc (S/CO 2.73) and negative HBeAg. Based on the positive anti-HBc result, quantitative HBV DNA testing was subsequently performed and revealed detectable HBV DNA with a viral load of 9.54 × 10 ³ IU/mL. The overall serological profile and interpretation of hepatitis B markers are summarized in Table 1*.*

**Table 1:** Interpretation of HBV markers.

**Marker**	**Result**
HBsAg	Negative
Anti-HBs	292.3 IU/L
Anti-HBc	Positive
HBeAg	Negative
HBV DNA	9.54 × 10 ³ IU/mL

Interpretation: The serological profile (HBsAg-negative, anti-HBc-positive, anti-HBs-positive) was initially suggestive of resolved HBV infection. However, detectable HBV DNA confirmed occult hepatitis B infection.

Abbreviations: HBsAg, hepatitis B surface antigen; anti-HBs, antibody to hepatitis B surface antigen; anti-HBc, antibody to hepatitis B core antigen; HBeAg, hepatitis B e antigen; HBV, hepatitis B virus;

Based on these findings, the patient was diagnosed with occult hepatitis B virus infection. Antiviral prophylaxis was initiated to reduce the risk of viral reactivation in the setting of future immunosuppressive therapy after kidney transplantation. According to the guidelines of the Vietnamese Ministry of Health, patients with HBsAg-positive or anti-HBc-positive indications for organ transplantation should start hepatitis B antiviral medication as soon as possible regardless of HBV DNA levels. Therefore, the patient was started on Tenofovir alafenamide 25 mg once daily, adjusted for end-stage renal disease and maintenance hemodialysis, and administered after dialysis sessions. Due to the lack of a suitable kidney donor, the patient declined proceeding with transplantation, continued on hemodialysis, and discontinued Tenofovir alafenamide 25 mg.

## Discussion

This case highlights an important diagnostic pitfall in hepatitis B screening. Despite negative HBsAg and high anti-HBs titers, HBV DNA testing revealed the presence of circulating virus, confirming occult hepatitis B virus infection [15]. Relying solely on HBsAg to exclude HBV infection is insufficient in high-risk populations. Previous studies have reported the presence of OBI among hemodialysis patients with negative HBsAg, with varying prevalence rates ranging from 3.8% to 6.0% depending on geographic region and diagnostic methods [[16], [17], [18]]. Currently in Vietnam, there are no large-scale statistical studies on the prevalence of OBI in the community and in hemodialysis patients. Vietnam is also a country with a high HBV prevalence rate, so the risk of missing OBI cases in clinical practice is possible [2]. OBI holds particular significance in pre-transplant assessment because prolonged post-transplant immunosuppression can trigger HBV reactivation, leading to elevated HBV DNA levels, the reappearance of HBsAg, and liver injury. In severe cases, HBV reactivation can result in severe hepatitis or liver failure, adversely affecting the patient's prognosis. Therefore, detecting OBI prior to transplantation plays a crucial role in risk stratification and in selecting strategies for monitoring and preventing post-transplant HBV reactivation [9].

Another notable feature of this case is the coexistence of a high anti-HBs titer and detectable HBV DNA. Although anti-HBs are generally considered protective, their presence does not necessarily indicate complete viral clearance. Persistence of covalently closed circular DNA within hepatocytes may allow low-level viral replication despite serological recovery [19]. Additionally, mutations in the HBV surface gene may alter the antigenic structure of HBsAg, leading to reduced detectability by standard assays [10]. Such immune escape variants may result in false-negative HBsAg results despite ongoing viral replication [20]. Impaired immune surveillance in patients with chronic kidney disease may further facilitate viral persistence.

A noteworthy aspect of this case is the relatively HBV DNA level (9.54 ×10^3^ IU/mL), which is higher than typically reported in occult hepatitis B infection. Although OBI is usually associated with very low or intermittently detectable viral loads, transiently higher HBV DNA levels may occur in certain clinical situations, particularly in immunocompromised individuals such as patients undergoing long-term hemodialysis. Current guidelines recommend screening transplant candidates using both HBsAg and anti-HBc testing, followed by HBV DNA testing in individuals with evidence of prior exposure [12,21]. However, implementation of these recommendations remains inconsistent in many healthcare settings, particularly in resource-limited regions [22,23].

Occult hepatitis B virus infection may be overlooked when screening relies solely on HBsAg and anti-HBs testing. This case illustrates a diagnostic pitfall in the pretransplant evaluation of patients with chronic kidney disease. All kidney transplant candidates should undergo anti-HBc screening as recommended by current guidelines. anti-HBc and HBV DNA testing should be considered in anti-HBc-positive individuals to identify occult HBV infection before transplantation.

## CRediT authorship contribution statement

**Bui Thai Huy Nguyen:** Writing – original draft, Investigation, Data curation, Conceptualization. **Hoang Phuong Tran:** Investigation, Data curation. **Nguyen Tra Uyen Ho:** Investigation, Data curation. **Thai Nguyen Ma:** Investigation, Data curation. **Tin Nghia Tran:** Investigation. **Quang Khai Tran:** Writing – review & editing. **Duy Linh Nguyen:** Writing – review & editing, Supervision, Conceptualization.

## Ethics declaration

Written informed consent to take part in the study and to publish the article has been obtained from all participants or their legal representatives. The privacy rights of participants have been observed.

This study was performed in compliance with relevant laws, regulatory frameworks and guidelines where the research took place. Ethics committee approval was not required under relevant laws and institutional guidelines. Ethics committee approval was not required for this single-patient case report in accordance with institutional requirements. Written informed consent was obtained from the patient for publication of the case report and accompanying clinical information.

This research follows the CARE guidelines and the CARE checklist.

## Informed consent

Written informed consent was obtained from the patient for publication of this case report and accompanying clinical information.

## Funding

None.

## Declaration of Competing Interest

The authors declare that they have no known competing financial interests or personal relationships that could have appeared to influence the work reported in this paper.
